# Layered Metal–Organic Chalcogenides: 2D Optoelectronics in 3D Self-Assembled Semiconductors

**DOI:** 10.1021/acsnano.4c18493

**Published:** 2025-03-26

**Authors:** Watcharaphol Paritmongkol, Zhifu Feng, Sivan Refaely-Abramson, William A. Tisdale, Christoph Kastl, Lorenzo Maserati

**Affiliations:** † Department of Materials Science and Engineering, School of Molecular Science and Engineering (MSE), Vidyasirimedhi Institute of Science and Technology (VISTEC), Rayong 21210, Thailand; ‡ Istituto Italiano di Tecnologia, Genova 16163, Italy; § Department of Molecular Chemistry and Materials Science, Weizmann Institute of Science, Rehovot 76100, Israel; ∥ Department of Chemical Engineering, Massachusetts Institute of Technology, Cambridge, Massachusetts 02139, United States; ⊥ Walter Schottky Institute, TUM School of Natural Sciences, Technical University of Munich, Garching 85748, Germany; # Munich Center for Quantum Science and Technology (MCQST), Munich 80799, Germany; ∇ Laboratorio Energia Ambiente Piacenza (LEAP), Piacenza 29121, Italy

**Keywords:** metal−organic chalcogenides, MOC, exciton, 2D semiconductors, low-dimensional hybrids, mithrene

## Abstract

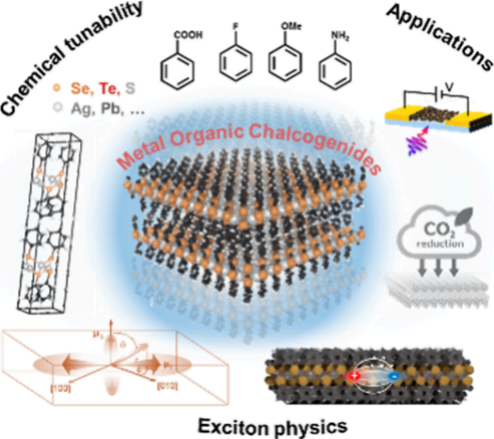

Molecular self-assembly offers an effective and scalable way to design nanostructured materials with tunable optoelectronic properties. In the past 30 years, organic chemistry has delivered a plethora of metal–organic structures based on the combination of organic groups, chalcogens, and a broad range of metals. Among these, several layered metal–organic chalcogenides (MOCs)—including “mithrene” (AgSePh)—recently emerged as interesting platforms to host 2D physics embedded in 3D crystals. Their combination of broad tunability, easy processability, and promising optoelectronic performance is driving a renewed interest in the more general material group of “low-dimensional” hybrids. In addition, the covalent MOC lattice provides higher stability compared with polar materials in operating devices. Here, we provide a perspective on the rise of 2D MOCs in terms of their synthesis approaches, 2D quantum confined exciton physics, and potential future applications in UV and X-ray photodetection, chemical sensors, and electrocatalysis.

The ability to control the atomic composition of inorganic materials with a percentage per billion accuracy and fabricate nanostructures in a top-down approach over macroscopic lengths paved the way for the (silicon) semiconductor era. At the same time, over the past 40 years, the development of bottom-up nanofabrication strategies (analogous to the ones developed by biological systems) has provided scientists access to precise nanoscale manipulation of organic materials by rational molecular design and subsequent self-assembly. Combining inorganic atomic constituents with good electrical properties and organic functional groups with known molecular chemistry, a bottom-up approach can open the door to subnanoscale precision in the fabrication of hybrid semiconductor device architectures. One of the most interesting possibilities of such a bottom-up approach is the exploitation of quantum physical phenomena realized in confined, low-dimensional architectures, such as 0D (quantum dots), 1D (quantum wires), and 2D (quantum wells) systems.^1^ Among such structures, hybrid 2D quantum wells are an intriguing platform for optoelectronic applications that are electrically contactable, show high photon absorption cross-section, exhibit relatively high charge mobility, and can be processed at scale with low-cost materials and techniques.^2^ While 2D metal halide perovskites are a widely recognized example of such hybrid quantum well materials, their operational instability and the presence of toxic lead in perovskite constituents, despite a widespread effort to find mitigation strategies, still hamper their practical use.^3−5^ In this Perspective, we highlight an alternative platform based on two-dimensional (2D) metal–organic chalcogenides (MOCs), which emerged in the last six years.^6−15^

Layered metal–organic chalcogenides (MOCs) are crystalline materials in which covalently bound inorganic 2D layers are sandwiched by organic spacer layers. Assembly of these layers by weak van der Waals interactions forms 3D bulk crystals, effectively composed of electronically decoupled 2D layers. These 2D hybrid quantum wells constitute a broadly appealing material platform touching fundamental aspects of material chemistry, solid state physics, in particular many-body optical phenomena, as well as optoelectronic applications (Figure 1). MOCs combine the chemical tunability of the inorganic backbone with the possibility of organic functionalization via the spacer layer (Figure 1a,b). Basic 2D MOCs consist of layers of metal atoms, such as Ag, arranged in tetrahedral coordination with chalcogen atoms, such as Se, which are also covalently bound to organic phenyl groups. For AgSePh, the phenyl rings are not interlocked, and their rotational freedom was thought to lead to multiple lattice configurations described by space groups *C*2/*c* and *P*21/*c*.^17^ Nevertheless, a recent study strongly supports the presence of the latter symmetry only.^18^ The hybrid lattice provides an effective 2D quantum confinement of the in-plane delocalized charge carriers, as evidenced in DFT calculations by the flat electronic dispersion in the out-of-plane direction and the anisotropic dispersion in the in-plane direction (cf. Electronic Structure and Exciton Anisotropy Section). 2D excitonic transitions (Figure 1c) govern the optical absorption and photoluminescence dynamics (cf. Exciton Dynamics Section), and they are efficiently coupled to lattice vibrations (Figure 1d and Exciton–Phonon Coupling Section). The energy of these optical transitions can be tuned considerably by changing the chalcogen of the lattice. While some pioneering works on the synthesis^17^ and photophysics^19,20^ of MOCs appeared already in the early 2000s, more recent synthetic approaches for fabricating scalable thin films^10,13,21,22^ and high-quality single crystals^6,12,23^ enabled in-depth studies of the peculiar 2D exciton physics in this material class.^6,7,24^ In particular, building upon the thin film fabrication of the silver phenylselenolate (AgSePh)—also known as “mithrene”—a prototypical member of the 2D MOCs, few groups reported peculiar room temperature stable, 2D-like excitonic properties,^24^ in-plane structural^18^ and optical anisotropy (Figure 1e),^6,25^ elemental-tunable excitons,^6,26^ strong exciton–phonon coupling,^14,15,27,28^ strong exciton–polariton coupling,^29^ and *p*-type transport behavior.^16,30^ Considering their ease of fabrication, high absorption cross-section, and tunable semiconducting behavior, possible optoelectronic and electrocatalytic applications are envisioned. So far, several proof-of-concept devices, focusing on photoconductors for UV^16^ and X-ray direct detection^31^ and flexible devices, have been demonstrated, reporting high sensitivity, low limit of detection, and promising operational stability (Figure 1f,g and Applications in Optoelectronic Devices Section). Applications are also emerging in the field of electrocatalysis for CO_2_ reduction reaction (CO_2_RR), demonstrating an high Faraday efficiency in the conversion of CO_2_ to CO with a good selectivity against the competing hydrogen evolution reaction (HER).^32^ Finally, MOCs have been recently utilized in molecular detection,^33^ gas sensing with ultralow detection limits,^34,35^ and as passivation precursors for improving the efficiency and stability of perovskite solar cells.^36^

**Figure 1:**
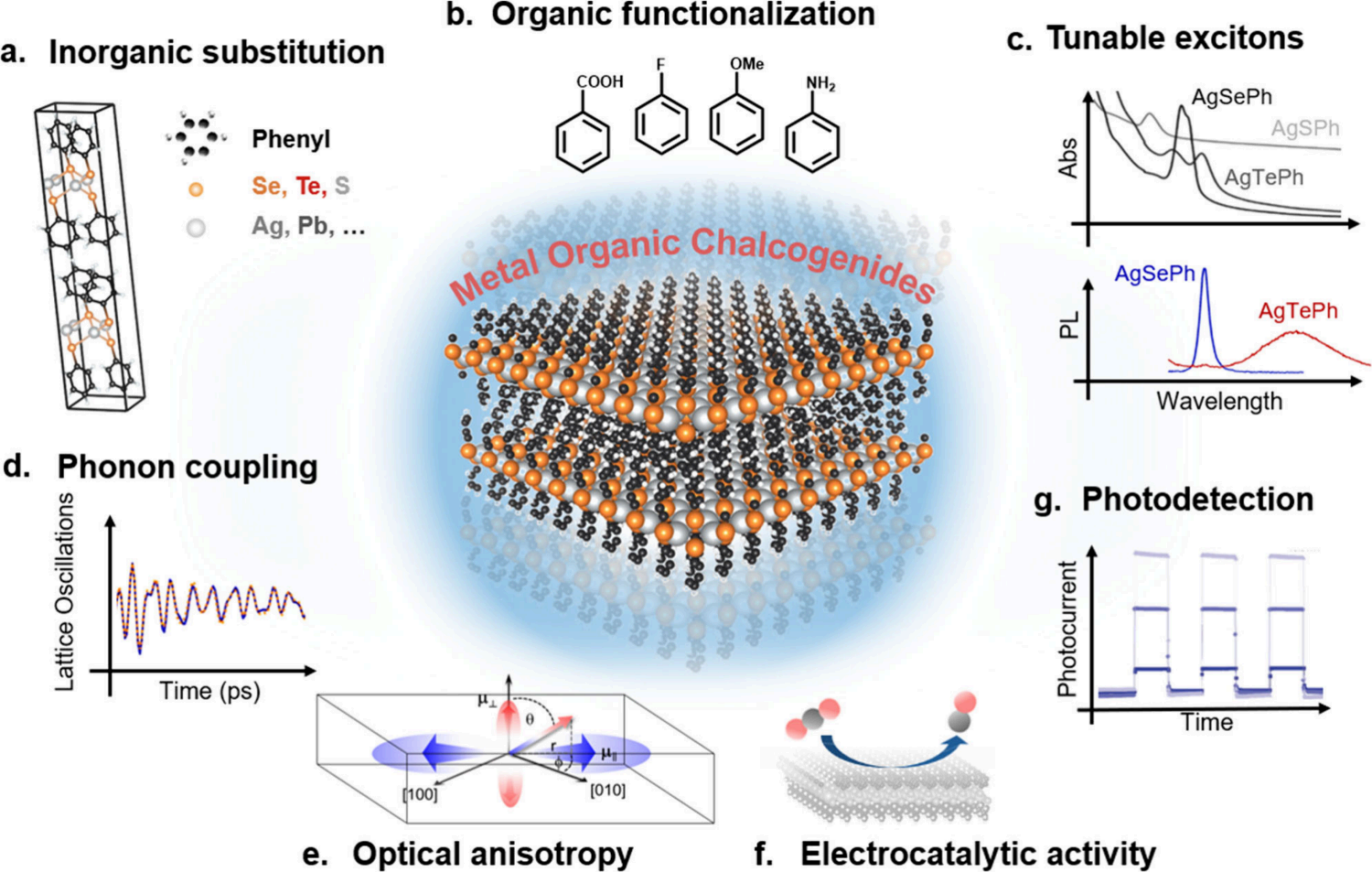
**Overview of current progress in MOC research.** The material platform allows addressing fundamental questions in chemistry, physics, and applications of organic/inorganic hybrid quantum well materials. (a, b) The prototypical 2D MOC unit cell allows for chemical tunability by (a) metal and chalcogen substitution and (b) organic group functionalization. (c) Optical absorption and photoluminescence spectra are tuned by the chalcogen atom in the MOC lattice. (d) Strong exciton–phonon coupling manifests as coherent phonon oscillations in transient absorption at picosecond time scales. (e) Structural anisotropy translates into strong optical anisotropy (both in-plane and out-of-plane). (f) Interaction with volatile molecular species yields chemosensitivity as well as electrocatalytic activity. (g) Flexible UV and X-ray MOC photodetectors with high responsivity were demonstrated. [Credits: a, Adapted with permission from ref (6). Copyright 2021 by the Royal Society of Chemistry; g, Reprinted with permission from ref (16). Copyright 2021 by the Royal Society of Chemistry.]

## Synthesis

The first synthesis of layered MOCs dates back to 1973 when a few organometallic derivatives of alkane- and arene-thiols were reported.^37^ In this seminal work, AgSePh was obtained from silver nitrate (AgNO_3_) and bis(phenyseleno)dibutyltin (Bu_2_Sn(SePh)_2_) in methanol:
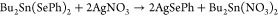
1Around the same time, MOCs with different metals (M) were also synthesized by reacting M(SePh)_3_ with metal halides.^38^ In 2002, the homoleptic and polymeric complex [Ag(SePh)]_**∞**_ was synthesized through the reaction of silver chloride (AgCl) and triphenylphosphine (PPh_3_) with either PhSe^–^ or PhSeS^–^.^17^ At this time, its crystal structure was first refined, confirming the 2D layered structure. Then, 16 years later, a different synthesis was introduced by the Hohman group, starting from an aqueous AgNO_3_ solution and a Ph_2_Se_2_ solution in toluene, leveraging the precursors’ immiscibility to reach product formation at the liquid–liquid interface.^10^ In this work, they discovered the narrow blue luminescence and excitonic property of AgSePh or “mithrene”, a prototypical example of the 2D MOC family, sparking a broader interest in the material platform and stimulating the development of additional synthetic routes to facilitate production and improve material quality.

**Figure 2:**
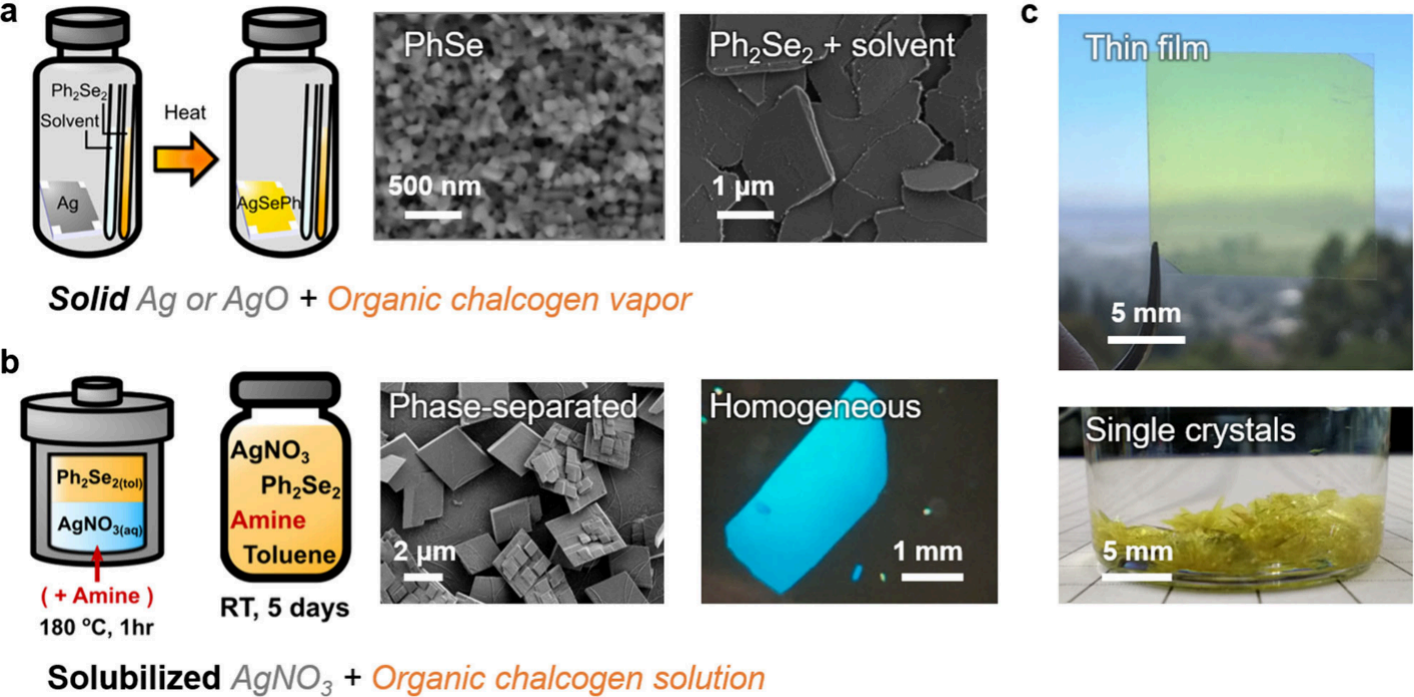
**Reported pathways for AgEPh syntheses, where E is a chalcogen atom and Ph is a phenyl group.** (a) Metal–organic reactions obtained via vapor attack (of PhSe or Ph_2_Se_2_ precursors) to Ag thin films. The addition of vaporized solvents can be used to further moderate the reaction and control the crystal size and morphology from nano- to microcrystalline films. (b) Solution syntheses from silver salts (e.g., AgNO_3_) either from the phase-separated liquid interface or as homogeneous mixtures yielding micrometer- to millimeter-sized isolated crystals. (c) Optimized synthesis protocols yield scalable thin films or large, isolated single crystals. [Credits: Reprinted with permission from refs (10, 12, and 13). Copyright 2018, 2021, and 2022 by the American Chemical Society. Reprinted with permission from refs (6 and 16). Copyright 2021 by the Royal Society of Chemistry.]

In general, the synthesis of AgSePh can be classified into two categories based on the precursors’ phases (Figure 2): (i) solid–gas reaction and (ii) solution-based synthesis. In the solid–gas reaction (Figure 2a), the metal sources are usually metals or metal oxides.^21,22^ The reaction proceeds via the interaction of these metal sources with vaporized organochalcogenide precursors (e.g., Ph_2_Se_2_ or PhSeH for the synthesis of AgSePh) and can be described as follows:
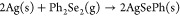
2or
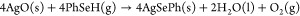
3The choices of the metal sources and organochalcogenide precursors have an influence on the reaction rate. When Ag and Ph_2_Se_2_ are used, the reaction takes days to complete due to the required oxidation/reduction steps. In contrast, when AgO reacts with PhSeH, the process can reach completion in 30 min. With this solid–gas reaction approach, wafer-scale nanocrystalline films can be synthesized from metal films. A benefit of this approach is the direct preparation of semiconductor films for subsequent device fabrication. Another is the low reaction temperature of ∼80–100 °C, which is attractive for electronic fabrication processes with low energy consumption. However, the grain sizes of the resulting AgSePh films are usually small, on the order of ∼200 nm (Figure 2a), which may inhibit the performance of AgSePh devices. Higher quality AgSePh films with micron grain sizes can be obtained by introducing solvent vapors.^13^ Incorporating dimethyl sulfoxide or propylamine (PrNH_2_) in the reaction vessel led to an increase of grain size to 5 μm (Figure 2a) and a higher photoconductivity due to a lower defect concentration. An issue of consideration for this approach is the use of thermal evaporators for metal precursor preparation, which may be attractive for industries but is less accessible among academic laboratories.

Another approach to AgSePh synthesis is solution-based. Reaction of solubilized silver salt with an organochalcogenide solution, either phase-separated or in a homogeneous mixture, produces micro- to millimeter-sized AgSePh crystals (Figure 2b). Phase separation can be easily achieved, e.g., by layering a solution of Ph_2_Se_2_ in toluene on top of an aqueous AgNO_3_ solution, resulting in the production of micro AgSePh crystals at the interface.^10^ The concentration of Ph_2_Se_2_ was found to weakly correlate to the thickness of the crystals, while the amorphous byproduct fraction was affected by the silver concentration and growth time. Higher reaction temperatures and hydrothermal conditions suppress the generation of the amorphous byproducts.^39^ The higher temperature and pressure also reduce the reaction time from days to hours and increase the crystal size from 2 to 5 μm. Even larger crystals can be obtained by introducing amines to the aqueous AgNO_3_ solution.^12^ Amine molecules are generally known to form silver–amine complexes, a key ingredient in Tollen’s reagent for detecting aldehyde functional groups in organic chemistry. Due to this complex formation, the reaction kinetics are slowed down, leading to improved crystallization and crystal quality, as evidenced by a larger crystal size of ∼10–100 μm and suppressed optical emission from midgap defect states.

The solution-based reaction can be performed in a homogeneous mixture as well as by mixing AgNO_3_ with PhSeH or Ph_2_Se_2_ in a water/alcohol solution.^32,40^ Amine addition to form silver–amine complexes is also applicable to this single-phase approach. By dissolving AgNO_3_ and Ph_2_Se_2_ in propylamine (PrNH_2_) or longer-chain amines, large millimeter-sized single crystals can be obtained.^12^ The method has proven successful for AgSePh and its analogs: AgSPh, AgTePh, and AgSePhMe.^12,18^ While crystallization still requires improvement in terms of generality, reliability, and speed, the two synthesis routes discussed above can already yield scalable thin films or large isolated single crystals (Figure 2c) suitable for different applications or experiments. On the scalability side, a microwave-assisted method was demonstrated, achieving a yield of 83 wt % in a few hours for AgSePh and AgSPh powder production from low-toxicity precursors.^32^ Finally, building on previous reports,^23,41,42^ a recent hot-injection synthesis has shown a scalability of up to 0.4 g per batch for the preparation of AgSePh nanocrystals.^30^

In addition to the synthesis development, several organic modifications of AgSePh have already been investigated. Functionalization at the para position of the phenyl ring to obtain biphenyl, para-methoxyphenyl, para-fluorophenyl, and para-alkylphenyl slightly shifts the photoluminescence emission peak while maintaining 2D structures.^12,24,43^ However, ortho functionalization and heterocyclic modification at this location lead to 1D and 0D structures with changes in their excitonic behaviors and luminescent properties.^44,45^

Considering the sulfur equivalent AgSPh, engineering of the organic para substituent to the SH group directly affects the electron conductivity inside MS(Ph–X) quantum wells, with M = Ag^+^, Cu^+^, and Au^+^ and X = −COOH, −F, −OCH_3_, −OH, and −NH_2_.^9^ Alternatively, fluorine can be used to tune the polarization of the phenyl ring, which barely impacts the optical properties while significantly changing the symmetry group of the layered structure.^46^

## Electronic Structure and Exciton Anisotropy

**Figure 3:**
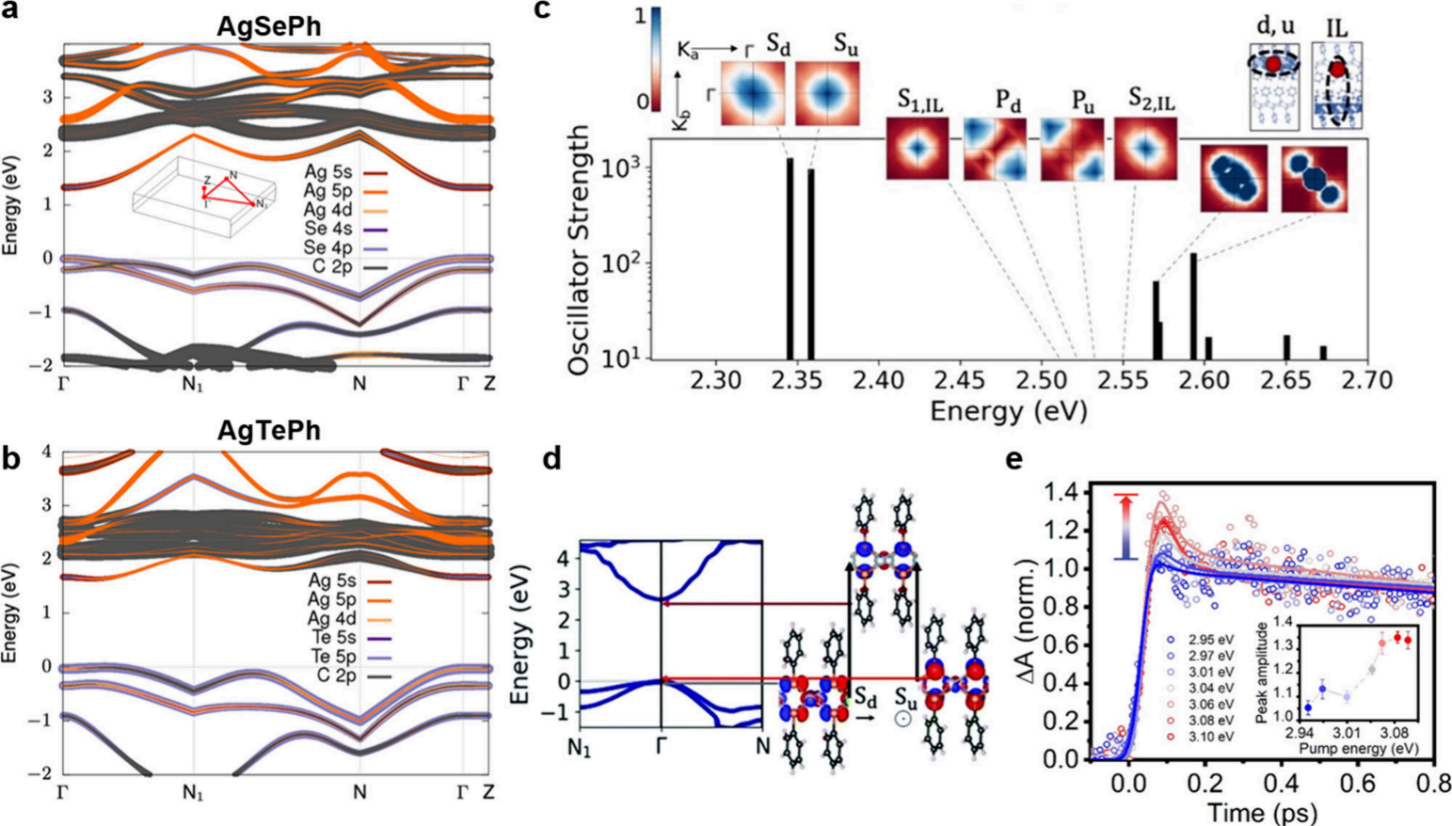
**Electronic structure and optical properties from first-principles.** The band structure calculated by DFT shows a direct quasi-particle gap at Γ for (a) AgSePh and (b) AgTePh. (c) *Ab initio* GW-BSE calculations of AgSePh predict multiple, strongly bound states of 2D intralayer exciton character. (d) Single-particle wave functions at the Γ point are localized to the inorganic planes. (e) Experimental observation of the onset of free carrier excitations and quasi-particle gap in AgSePh. The ultrafast carrier dynamics accelerate for excitation above the quasi-particle gap of 3.05 eV. [Credits: a, b, Reprinted with permission from ref (7). Copyright 2022 by the American Chemical Society; c–e, Reprinted with permission from ref (6). Copyright 2021 by the Royal Society of Chemistry.]

Figures 3a and 3b depict the electronic band structures of AgSePh and AgTePh calculated using approximate DFT with the PBE functional and semiempirical DFT-D2 correction.^7^ Within the direction of the inorganic plane, AgSePh is a direct gap semiconductor with the band gap located at the Γ-point and effective electron and hole masses on the order of 0.5–1*m*_e_, which are comparable to other 2D vdW semiconductors, such as WS_2_ with an effective hole mass of 0.4–0.5*m*_e_.^47^ Toward the application of AgSePh in electronic devices, a relatively high hole mobility of 4.88 × 10^–2^ cm^2^·V^–1^·s^–1^ in nanocrystalline AgSePh films has been very recently observed using the space-charge limited current (SCLC) technique.^30^ This value is competitive with SCLC-derived hole mobility values of commonly used inorganic, thin-film hole conductors, such as polycrystalline CuSCN films ((2.6–6.4) × 10^–2^ cm^2^·V^–1^·s^–1^)^48,49^ and NiO_*x*_ nanocrystal films ((0.8–3.2) × 10^–2^ cm^2^·V^–1^·s^–1^).^50,51^

At the band edges, the inorganic AgSe layers contribute predominantly to the electronic structure with orbital character mostly from Ag 4d and Se 4p; the conduction band minimum additionally features Ag 5s character.^6,7^ Along the Γ–Z direction, the dispersion is flat, which is consistent with an effective electronic decoupling of the van der Waals stacked layers via the organic phenyl groups and the corresponding quantum confinement along this direction into the inorganic planes.

It is worth noting that the electron density at the CBM also contains a small orbital contribution from the nearest C atoms suggesting tunability by modification of the organic group in addition to the optical tunability derived by the chalcogen choice or by alloying different chalcogens in the same crystal (AgEPh; E = S, Se, Te) that very recently allowed continuously tunable optical transitions to be achieved.^26^

While AgSePh exhibits simple parabolic dispersions around Γ (Figure 3a), the electronic structure of AgTePh reveals a saddle point behavior near Γ, in both the conduction and valence band edges (Figure 3b). Although the character of the quasi-particle gap can still be considered as direct (the difference between the direct and indirect gaps is less than 11 meV), the saddle points and associated van Hove singularities may enhance carrier interactions and facilitate the excitonic self-trapping found experimentally. However, a thorough understanding of the peculiar optical properties of AgTePh is still lacking.

In general, 2D materials often require the inclusion of excitonic effects for a proper theoretical description of their light–matter interactions and carrier dynamics. In this context, the optical properties of AgSePh were investigated by *ab initio* GW-BSE calculations. The calculations predict multiple exciton states (Figure 3c) below the quasi-particle gap (theoretical value of 2.7 eV). Consistent with the orbital character of the band edges (Figure 3d), the lowest exciton states S_d_ and S_u_ are strongly confined to the AgSe planes (2D intralayer excitons), and they appear around 2.35 eV, corresponding to a theoretical exciton binding energy of 350 meV, which is comparable to monolayer transition metal dichalcogenides.^52−54^ Experimentally, the quasi-particle gap was determined to be 3.05 eV by identifying the onset of free carrier relaxation at short time scales^6,55^ after a detuned, fs-pulse excitation (Figure 3e). This translates into exciton binding energies of 380 (X_1_), 310 (X_2_), and 180 meV (X_3_), in reasonable agreement with the theoretical predictions.

**Figure 4:**
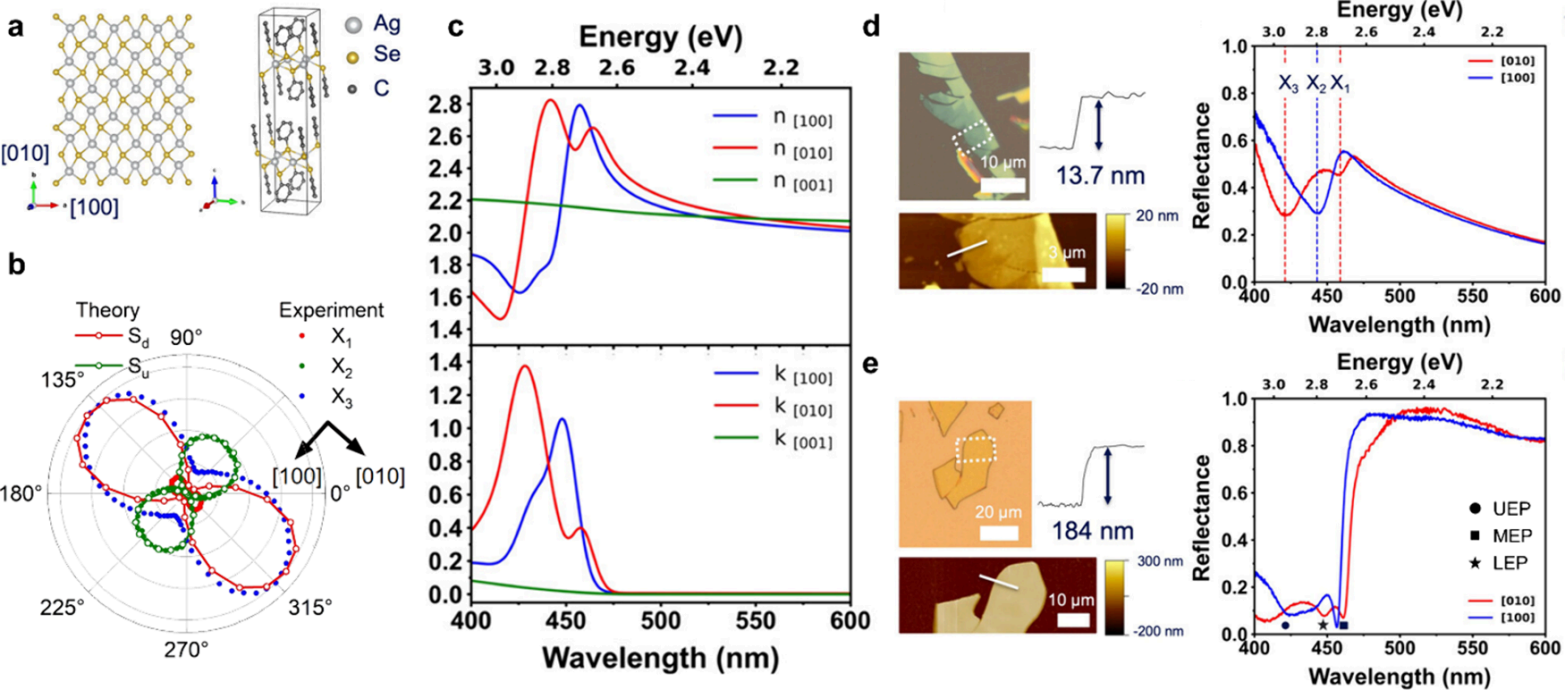
**In-plane anisotropy of single-crystal AgSePh sheets.** (a) Top view of the inorganic plane of AgSePh highlighting the broken symmetry between the [010] and [100] directions. (b) Comparison of the angular dependence of the theoretically predicted and experimentally measured anisotropic, excitonic absorbance. (c) Refractive index and absorption coefficient determined from Mueller matrix ellipsometry. (d, e) Polarization-dependent reflectance spectra on a thin (14 nm) (d) and thick (184 nm) (e) AgSePh crystal. For thin films, the reflectance reveals anisotropic exciton resonances X_1_, X_2_, and X_3_. For thick films, self-hybridization of exciton and photon results in the emergence of an upper exciton-polariton (UEP), a middle exciton-polariton (MEP), and a lower exciton-polariton (LEP). [Credits: a, c–e, Reprinted with permission from ref (25). Copyright 2024 by the American Chemical Society; b, Reprinted with permission from ref (6). Copyright 2021 by the Royal Society of Chemistry.]

In addition to the apparent difference between in-plane and out-of-plane optical properties due to the layered structure of AgSePh, the in-plane anisotropy of its lattice (Figure 4a) directly translates into a significant birefringence of the exciton resonances in single-crystal samples. The in-plane optical anisotropy of the lowest exciton states was theoretically predicted and experimentally verified by their linearly polarized absorbance (Figure 4b).^6^ Very recent studies further exploited the anisotropy of the exciton states to demonstrate strong birefringence and crystal-axis-dependent attenuation at the exciton transitions (Figure 4c), which was coined “linear dichroism” in exfoliated and transferred AgSePh crystals.^25^ As a consequence of their comparably large index (*n* > 2.5 for the exciton resonances), MOCs support the self-hybridization of excitons and photons for thick single-crystal films without any additional cavities. While thin films show characteristic resonances at the energies of the X_1_, X_2_, and X_3_ excitons (Figure 4d), thick films show resonances consistent with the formation of exciton-polaritons (Figure 4e). The anisotropy of the excitons results in a giant anisotropy and degree of linear polarization of the exciton-polaritons as well.^24^ The latter renders MOCs a polaritonic, birefringent materials platform for polarization-sensitive applications in the short wavelength regime.^56^

## Exciton Dynamics

**Figure 5:**
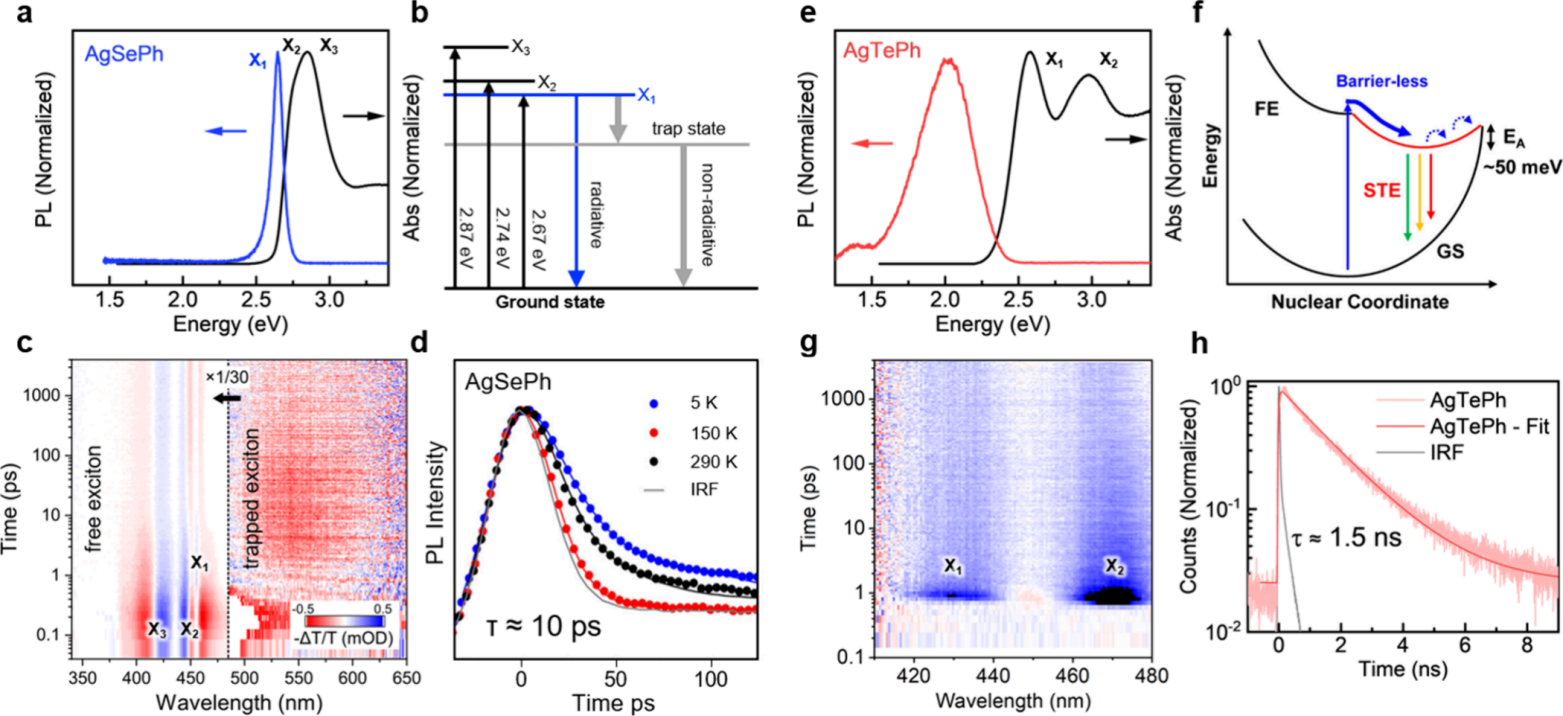
**Optical properties and carrier dynamics of AgSePh and AgTePh.** (a) Room temperature absorption and photoluminescence spectrum of AgSePh. (b) Schematic level diagram with three free exciton states (X_1_, X_2_, and X_3_) and one trapped exciton state. The radiative emission occurs from the lowest free exciton state (X_1_) with a competing nonradiative decay via the trapped exciton state. (c) Transient-absorption dynamics of short-lived (picoseconds) free and long-lived (nanoseconds) trapped excitons. (d) Time-resolved luminescence of AgSePh with ps-lifetime. (e) Absorption and photoluminescence spectrum of AgTePh. (f) Schematic of the intrinsic self-trapping of free excitons (FEs) resulting in broadband, efficient radiative recombination via a self-trapped exciton state (STE). (g) Transient-absorption dynamics of AgTePh showing fast decay of the free excitons (X_1_, X_2_) due to self-trapping. (h) Time-resolved luminescence of AgTePh with ns-lifetime. [Credits: a, e, f, h, reprinted with permission from ref (7). Copyright 2022 by the American Chemical Society; c, Reprinted with permission from ref (14). Copyright 2022 by the American Chemical Society; d, Reprinted with permission from ref (24). Copyright 2021 by the American Chemical Society.]

The optical properties have been principally studied by using steady-state and time-resolved absorption and photoluminescence. Figure 5 contrasts the optical properties and exciton dynamics of AgSePh and the chalcogen-substituted compound AgTePh. The absorption spectrum (Figure 5a) of AgSePh contains multiple sharp excited states (labeled X_1_, X_2_, and X_3_). Radiative recombination occurs via the lowest excited state X_1,_ resulting in a narrow, blue emission line at 2.65 eV (468 nm) with a full-width-at-half-maximum (fwhm) of around 10 meV at ambient temperature.^6,7,10,14,24^ However, the quantum yield of the free emission in AgSePh was consistently found to be below 1%,^7,14^ even at cryogenic temperatures, which implies the existence of a competing nonradiative pathway, such as a trapped exciton state (Figure 5b). Time-resolved, transient absorption revealed indeed very fast decay of the free exciton population (on the order of a few ps) and delayed emergence of a photoinduced subgap state (Figure 5c).^14^ In AgSePh, the origin of such a photoinduced subgap absorption is still not conclusively resolved, as experimental evidence is consistent with both an extrinsic mechanism, i.e., trapping at defect sites,^12^ and an intrinsic mechanism, i.e., self-trapping of excitons.^14^ Independent of their exact physical origin, the subgap states in AgSePh provide efficient trapping and nonradiative recombination channels, such that the observed picosecond-luminescence dynamics (Figure 5d) and low quantum yield observed must be attributed to the dominant nonradiative recombination of excited states.

Interestingly, the excited state dynamics change completely in the sister compound AgTePh. The absorption spectrum of AgTePh displays multiple, yet rather broad, excited states, and the emission spectrum shows a broad continuum between 1.5 and 2.2 eV, significantly below the absorption onset at 2.5 eV (Figure 5e). Nevertheless, the quantum yield of AgTePh approaches 100% at low temperatures, in stark contrast to the low quantum yield seen in AgSePh. To explain the broad and efficient subgap emission in AgTePh, a barrierless self-trapping process was proposed (Figure 5f). In this picture, strong exciton–phonon coupling induces lattice deformation, resulting in efficient localization of excitons into a self-trapped state with an estimated escape barrier of 50 meV.^7^ At low temperatures, the latter is deep enough to prevent the detrapping of carriers. Then, radiative recombination from the self-trapped state into the ground state occurs via a continuum of possible transitions, as can be understood in a generalized coordinate representation (Figure 5f), also qualitatively explaining the spectrally broad luminescence of AgTePh. The transient absorption (Figure 5g) and luminescence dynamics (Figure 5h) support this picture with a fast picosecond decay of the free exciton population and a longer, radiative nanosecond decay of the trapped exciton population.

## Exciton–Phonon Coupling

**Figure 6:**
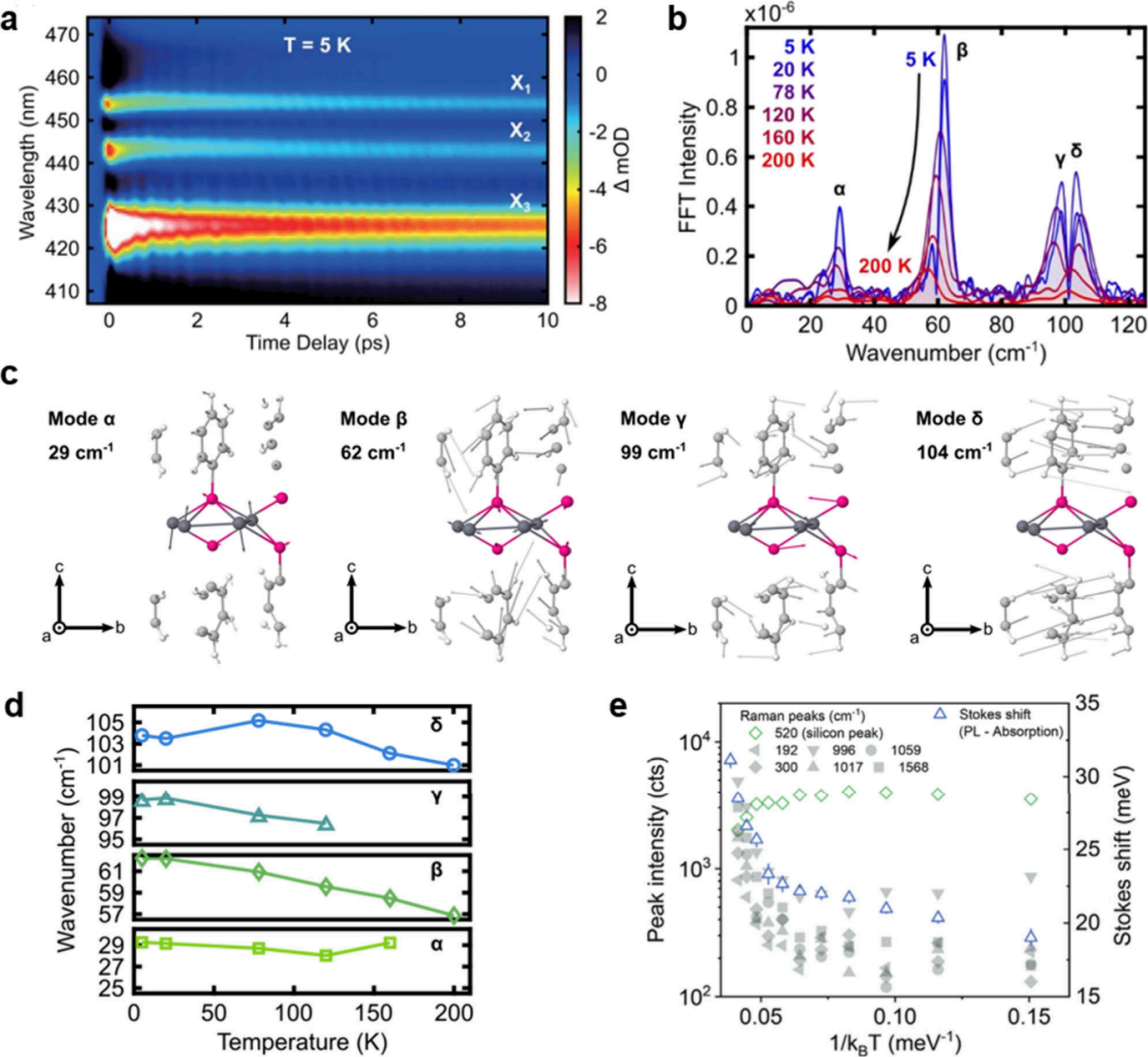
**Coherent dynamics and exciton–phonon interaction.** (a) Transient absorption spectrum at 5 K, highlighting exciton dynamics with clear phonon oscillations. (b) Fast Fourier Transform (FFT) of the transient absorption signals at the exciton energies up to 200 K. The peaks represent different low-energy phonon modes (Raman active). These modes are labeled as α, β, γ, and δ and are linked to the Raman modes calculated by DFT (c). (d) Shift of phonon modes with temperature. (e) Intensities of Raman modes at higher energy gray symbols show an unexpected suppression at low temperature that nevertheless positively correlates with the observed Stokes shift (blue triangles). The silicon Raman mode (green squares) is shown for comparison. [Credits: a–d, Reprinted with permission from ref (27). Copyright 2024 by Elsevier Inc.; e, Reprinted under the terms of CC BY 4.0 from ref (15). Copyright 2023 The Authors.]

Although AgSePh exciton wave functions mostly concentrate on the inorganic Ag–Se core, their phonon modes spread into the organic ligands, yielding complex and hybrid vibrational landscapes. Exciton–phonon coupling studies by resonant impulsive stimulated Raman scattering reveal three bleach features corresponding to excitons X_1_, X_2_, and X_3_ (Figure 6a). FFT analysis of the oscillatory signals over these bleach peaks at 5 K yields four prominent vibrational modes at 29.3, 62.1, 98.7, and 103.7 cm^–1^ (labeled as α, β, γ, and δ, respectively) in the low-frequency region (Figure 6b).^27^ The atomic displacements of these vibrational modes from density functional perturbation theory calculations show compound motions on both the inorganic core and organic ligand (Figure 6c). Correlation between experiment and simulation suggests that Ag atoms are displaced in the *z*-direction and that the change in Ag–Se interatomic bond spacing contributes the most to exciton–phonon coupling. Further investigation of the exciton–phonon coupling by low-temperature photoluminescence peak splitting, temperature-dependent photoluminescence peak shifting, and temperature-dependent photoluminescence line width broadening reveals the γ mode to most strongly influence the emission properties of AgSePh. Furthermore, temperature-dependent frequency shifts of the vibrational modes in AgSePh (Figure 6d) highlight a relatively high phonon anharmonicity of this hybrid material compared with all-inorganic solids. Additionally, an unusual temperature dependence is observed in the high-frequency Raman peak intensities (Figure 6e). These vibrations correspond mostly to C–C stretching and bending,^21^ and they are suppressed at lower lattice temperatures,^15^ in opposition to the standard intensity trend of the substrate peak from Si and of the AgSePh low energy anti-Stokes peaks (Figure 6b and ref (15)).^27^

## Applications in Optoelectronic Devices

**Figure 7:**
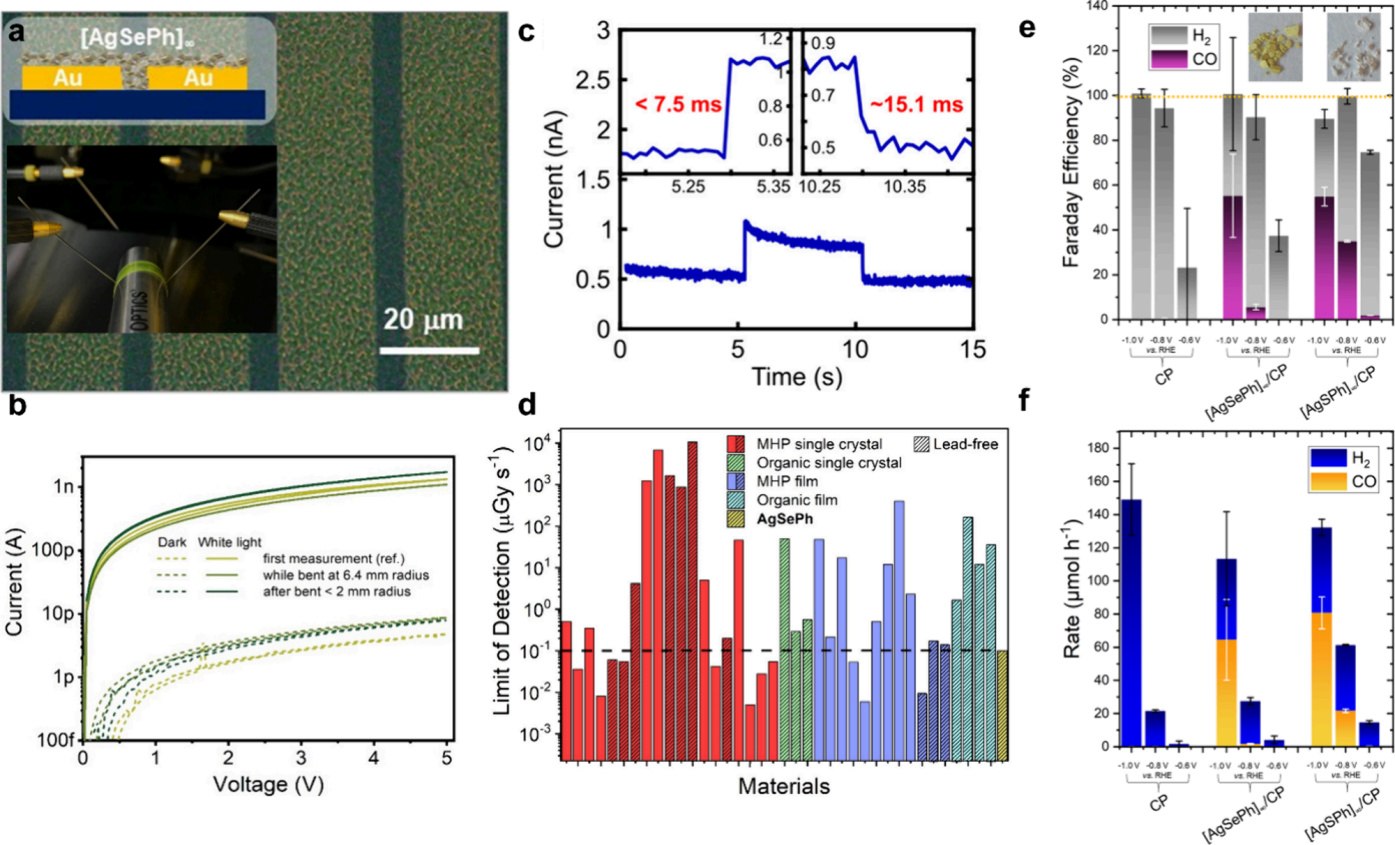
**Optoelectronic and photocatalytic applications of MOCs.** (a) Optical microscope image of a flexible, planar UV photodetector based on AgSePh synthesized on interdigitated metal electrodes supported on a polyethylene naphthalate substrate. Inset: schematic of the device layers and photograph of the electrical measurement under bending. (b) Electrical characteristics under light (solid lines) and dark (dashed lines) of the flexible device presented in (a). (c) Photocurrent rise and fall times of a AgSePh detector under blue light illumination. (d) The X-ray photocurrent response limit of detection (LoD) reported for different hybrid materials tested in recent years as X-ray direct detector active layers, highlighting the AgSePh-based device outperforming most competitors. Electrocatalytic performance of MOC-based devices for (e) CO_2_ to CO conversion (namely, CO_2_RR) illustrated in terms of Faraday efficiency and (f) CO/H_2_ production rate. [Credits: a, b, Reprinted with permission from ref (16). Copyright 2021 by The Royal Society of Chemistry; c, Reprinted with permission from ref (13). Copyright 2022 by the American Chemical Society; d, Reprinted under the terms of CC BY 4.0 from ref (31). Copyright 2023 The Authors; e, f, Reprinted under the terms of CC BY 4.0 from ref (32). Copyright 2023 The Authors.]

Layered metal–organic chalcogenides have already been used to demonstrate possible applications such as photodetectors (in the near visible and high energy range) and electrocatalyzers (Figure 7). For instance, a sensitivity of 0.8 A W^–1^ at a wavelength of 370 nm was reported for a AgSePh near-UV flexible photodetector in a planar device geometry (Figure 7a,b), which compares favorably with other 2D materials-based detectors (see ref (16) and references therein). The response time was measured to be on the order of 10–25 ms (Figure 7d).^13^ Reference (16) discusses the transport mechanism by the trapping of photogenerated holes and their subsequent release at recombination centers that saturate at medium light intensities (above 1 μW cm^–2^). The same planar device architecture was used to demonstrate a working coplanar direct X-ray photoconductive detector.^31^ The device was again fabricated on a flexible polyethylene naphthalate (PEN) with micropatterned gold electrodes covered by *in situ* synthesized AgSePh. At 20 V the limit of detection (LoD) was 100 ± 30 nGy s^–1^, a value that is competitive with most other metal halide perovskite and organic semiconductor detectors (Figure 7d). By varying the radiation dosage rates, the sensitivity per unit area was calculated to be 180 ± 10 μC Gy^–1^ cm^–2^, matching the performances of full-organic and hybrid perovskite thin-film detectors and surpassing the sensitivity of a-Se and poly-CZT detectors. The AgSePh detector performances were reported to be unaltered after six months of storage in dark ambient conditions.

Interestingly, MOCs also show potential for electrochemical applications. In this context, syngas (a mixture of CO and molecular hydrogen) production via MOC-mediated electrocatalytic reduction of CO_2_ was reported. AgSPh showed a good Faraday efficiency of 45% for CO and 55% for H_2_ (Figure 7e), with production rates up to 81 μmol h^–1^ (Figure 7f).^32^ The same study also demonstrated the stability of MOCs in various solvents, temperature ranges, and pH media as well as upon light irradiation. The same materials have also been tested for photocatalytic water splitting when coupled with TiO_2_, reporting up to 2.5 μmol h^–1^ of H_2_ production under UV light,^57^ opening an interesting route for their use in electrocatalysis.

In the molecular detection field, a high-accuracy and tunable metabolic molecular diagnosis platform to detect saccharides with a molecular weight range from 180 to 828 Da based on Cu(SPh–COOH)-assisted laser desorption/ionization mass spectrometry was reported.^33^ Different layered MOCs have already been utilized for gas sensing. Ag(SPh–NH_2_)^58^ and PbBDT (BDT = 1,4-benzenedithiolate)^34^ were reported to have the lowest limit of detection (0.9 and 0.51 ppb, respectively) among all the 2D chemresistive materials with high selectivity.

Finally, Cu-(4-mercaptophenol) MOC was successfully applied as a passivator precursor for creating a Cu_2_S extraction layer in a metal halide perovskite solar cell attached to the Pb defect sites. The passivation yielded a 1.8% efficiency net increase in parallel with remarkable stability improvements regarding exposure to humidity, heat, and light.^36^

## Challenges and Outlooks

While recent advances unveiled the potential of this versatile material platform, metal–organic chalcogenides are still underexplored from synthetic chemistry, physical chemistry, optical, and device perspectives. Below, we provide a brief discussion of the existing challenges and potential outlooks.

From a material chemistry standpoint, the organic spacer groups introduce a powerful element to modify and direct the reaction pathway, self-assembly, final structure, and ultimately the optoelectronic functionality of the synthesized materials. The various synthetic pathways developed in recent years have enabled a level of material tunability that was previously unattainable. The vapor-phase tarnishing approach is particularly interesting for scalable and sustainable device fabrication due to the low reaction temperature required. However, a problem with this approach is the presence of pinholes in the resulting films caused by the structural change from metal/metal oxide precursors to MOCs. Additionally, the film quality needs to be further improved beyond the existing performance achieved via solvent vapor introduction^13^ and compatibility with the existing device fabrication process and integration with inorganic semiconductors, on top of which existing technologies are based, requires rigorous testing and development. Some of these problems may be solved by altering the precursor chemistry and reaction conditions. Existing approaches that are successful for other thin film semiconductors, such as perovskites and copper thiocyanate, may be applicable to solve such problems.

On the solution-based synthesis, we have seen some progress, especially in terms of the reaction speed and scalability. Existing methods can produce microcrystals of MOCs in just an hour, but the size distribution, in particular toward nanoscale crystals, is still difficult to control.^32,39^ The smallest size of nanocrystals achievable so far is 30 nm, which is still too large to see the quantum confinement effect along the lateral dimension of the 2D MOCs.^43^

Production of large and high-quality MOCs is still a challenge that often inhibits structural determination, intrinsic property investigation, and high-performance devices. While their resistance to solvents is beneficial for the material, it hampers, at the same time, the implementation of solution-based recrystallization techniques, such as cooling-induced, slow-evaporation, and antisolvent-diffusion recrystallization. Existing crystallization methods, such as amine-assisted crystallization, rely on slowing down the reaction kinetics of the starting materials to favor the growth and ripening of large single crystals over the nucleation of new crystals. Despite the amine-assisted crystallization method being tested on four MOC derivatives (AgSePh,^12^ AgSePhMe,^12^ and AgSePy^45^), novel crystallization techniques to improve reproducibility and generalizability need to be developed. Finding a good solvent system or an alkahest may be crucial toward enabling such techniques.^59−61^

While a significant effort has been dedicated to property tuning of MOCs via metal, chalcogen, and organic selections, these efforts are still concentrated on a few members and structures.^9,34,62−66^ For example, varying metal constituents focused mostly on coinage metals (Cu, Ag, and Au) due to their clear similarity. Even though a few reports demonstrated MOCs with Pb, Sb, Sn, and In as constituents, novel MOCs with other metals are still waiting to be discovered and explored. Similarly, the choice of organic components being investigated so far is limited to simple linear alkyl chains^64^ and monofunctionalized benzene rings.^9,12,63^ Efforts to develop complex organic structures, such as branched chains, extended conjugated systems, and chiral structures, may expand the mechanical, optical, electrical, and thermal properties of MOCs beyond the existing sets. DFT calculations combined with machine learning and AI technologies may offer efficient pathways to design these novel MOCs.

From a material science perspective, a covalently bonded MOC lattice offers a more robust alternative to the polar 2D perovskites. Although MOCs are stable under ambient conditions and can withstand laser excitation to some degree, long exposure to intense laser illumination still leads to photobleaching, limiting photophysical studies and consistent device operation. The photodegradation may be the result of photogenerated heat related to the low PLQY of the material or caused by other processes. Investigation of the photodegradation mechanism can provide useful information for developing mitigation strategies.

Regarding MOC material physics, a detailed understanding of the excited state energetics and dynamics will be essential for controlling and exploiting strong light–matter interactions. While AgSePh has a narrow blue luminescence in the blue region, its photoluminescence quantum yield is still too low to be used in efficient optoelectronic devices. Understanding the origin of defects and developing improvement strategies are the keys to solving this challenging problem. Due to the structural similarity with TMDs, previously proven approaches for TMDs, such as chemical treatment, electrical control, and strain engineering, with some adaptation, may alter the photophysical properties of MOCs, boosting their quantum yield to near unity, as exemplified by the case of AgTePh. Furthermore, the relative “softness” of these materials, compared to traditional inorganic semiconductor platforms, deserves particular attention since it may couple lattice deformations, or phonons, with the excited optical states, or excitons.^67^ Although such exciton–phonon interactions are a challenge for energy-efficient light emission, they are also an opportunity for tuning light–matter interaction in these solids, tailoring photo down-conversion and fast on/off optical switching.^15,27,28^ While a basic understanding of several excitonic phenomena in these 2D hybrids is underway,^68^ an ultimate consensus on the interpretation of their optical transitions, which are heavily affected by the aforementioned exciton–phonon coupling, is still missing.^68^

From an application point of view, several proof-of-concept studies are already exploring the most promising application pathways, including photodetection, chemical sensing, electroreduction, and passivation layers in hybrid photovoltaic devices.^16,36,57,58^ However, addressing the material challenges illustrated above will be the key to realizing the promise of MOCs in optoelectronics, catalysis, and photonic applications.
